# Diagnosis and treatment of meralgia paresthetica between 2005 and 2018: a national cohort study

**DOI:** 10.1007/s10143-023-01962-0

**Published:** 2023-02-13

**Authors:** Benn Schönberg, Mareen Pigorsch, Doerte Huscher, Shlomo Baruchi, Jennifer Reinsch, Anna Zdunczyk, Christoph Scholz, Ann-Kathrin Uerschels, Nora F. Dengler

**Affiliations:** 1 Vertebral Spine Center Berlin, Breite Straße 46/47, 13187 Berlin, Germany; 2 Institute of Biometry and Clinical Epidemiology, Charité – Universitätsmedizin Berlin, Corporate Member of Freie Universität Berlin and Humboldt-Universität Zu Berlin and Berlin Institute of Health, Charitéplatz 1, 10117 Berlin, Germany; 3 Department of Neurosurgery, Charité – Universitätsmedizin Berlin, Corporate Member of Freie Universität Berlin, Humboldt-Universität Zu Berlin and Berlin Institute of Health, Charitéplatz 1, 10117 Berlin, Germany; 4 Department of Neurosurgery, Faculty of Medicine, Medical Center, University of Freiburg, Breisacher Str. 64, 79106 Freiburg, Germany; 5 Department of Neurosurgery, Universitätsklinikum Essen, Hufelandstraße 55, 45147 Essen, Germany; 6 Department of Neurosurgery, Charité - Universitätsmedizin Berlin, Hindenburgdamm 30, 12203 Berlin, Germany

**Keywords:** Meralgia Paresthetica, Lateral femoral cutaneous nerve, Neurolysis, Neurectomy, Pain in the anterolateral thigh, Surgical treatment of nerve compression

## Abstract

The prevalence of meralgia paresthetica (MP), which is caused by compression of the lateral femoral cutaneous nerve (LFCN), has been increasing over recent decades. Since guidelines and large-scale studies are lacking, there are substantial regional differences in diagnostics and management in MP care. Our study aims to report on current diagnostic and therapeutic strategies as well as time trends in clinical MP management in Germany. Patients hospitalized in Germany between January 1, 2005, and December 31, 2018, with MP as their primary diagnosis were identified using the International Classification of Disease (ICD-10) code G57.1 and standardized operations and procedures codes (OPS). A total of 5828 patients with MP were included. The rate of imaging studies increased from 44% in 2005 to 79% in 2018 (*p* < 0.001) and that of non-imaging diagnostic studies from 70 to 93% (*p* < 0.001). Among non-imaging diagnostics, the rates of evoked potentials and neurography increased from 20%/16% in 2005 to 36%/23% in 2018 (*p* < 0.001, respectively). Rates of surgical procedures for MP decreased from 53 to 37% (*p* < 0.001), while rates of non-surgical procedures increased from 23 to 30% (*p* < 0.001). The most frequent surgical interventions were decompressive procedures at a mean annual rate of 29% (± 5) throughout the study period, compared to a mean annual rate of 5% (± 2) for nerve transection procedures. Between 2005 and 2018, in-hospital MP care in Germany underwent significant changes. The rates of imaging, evoked potentials, neurography, and non-surgical management increased. The decompression of the LFCN was substantially more frequent than that of the LFCN transection, yet both types of intervention showed a substantial decrease in in-hospital prevalence over time.

**Supplementary Information:**

The online version contains supplementary material available at 10.1007/s10143-023-01962-0.

## Introduction

Meralgia paresthetica (MP) is the compression syndrome of the lateral femoral cutaneous nerve (LFCN) resulting in numbness and/or painful dysesthesia of the anterolateral thigh [1–4]. For a long time, it was considered a rare disease [5, 6], but, beginning in the 1990s, a substantial increase in its prevalence has been observed, most likely due to growing rates of obesity and diabetes mellitus (DM) [7–9].

In the absence of high-quality clinical trial data on MP, guidelines are lacking. Care for MP is determined predominantly by surgeon preference and experience [10, 11]. Most therapists agree that the diagnosis of MP is based primarily on clinical examination and patient history. To rule out a lumbar radiculopathy as a differential diagnosis, a spinal MRI is recommended. Neurophysiologic work-up may include LFCN conduction studies or somatosensory evoked potentials [12–14].

Treatment options include local injections, open neurolysis or neurectomy, and various neuro-modulative approaches [3, 15–25]. If surgical therapy is necessary, a variety of techniques exist. Some groups prefer decompressive techniques with nerve preservation or even transposition while others recommend neurectomy of the LFCN [26–28]. A recent meta-analysis found slightly superior pain relief and lower rates of revision procedures for neurectomy, compared to decompressive procedures [29].

Large-scale evidence on the choice of diagnostic and therapeutic modalities in MP is lacking. The assessment of current practice, potential variations, and time trends has never been performed. We designed a national study aiming to report current practice in the diagnostic and therapeutic management of patients hospitalized for MP in Germany and time trends between years 2005 and 2018.

## Materials and methods

### Study setting and data acquisition

In 2005, Germany had 82,437,995 inhabitants, compared to 83,019,213 in 2018 [10]. The German health system is mainly funded by the government. About one-third of all hospitals are run by private companies [11]. Data on all patients hospitalized in Germany between 2005 and 2018 with International Classification of Diseases (ICD-10) code G57.1, which represents the diagnosis of MP, were provided by the German Federal Statistical Office (GFSO) for every 2nd year as well as 2018 and were included in the analysis. Ethical approval for this study was granted by local authorities (EA 1/275/20). Inclusion criteria were the main diagnosis of MP represented by the ICD code G57.1. Patients hospitalized with MP as their main diagnosis in years 2005, 2007, 2009, 2011, 2013, 2015, 2017, and 2018 entered the final analysis. The ICD10 main diagnosis refers to the diagnosis that is the cause for hospitalization. Procedures are coded according to the German operations and procedures codes (OPS). An unlimited number of procedures can be assigned to a single patient. Different chapters describe the procedure type, such as chapter 1—diagnostic procedures, chapter 3—imaging methods, chapter 5—surgical procedures, chapter 6—specific drug applications, chapter 8—nonsurgical therapeutic procedures, and chapter 9—additional procedures. Outpatient data on MP were not included in the analysis as they are not available through the GFSO.

### Statistical analysis

Rates for diagnostics and procedures were calculated relative to patients hospitalized for MP. The presence of systematic time trends was investigated with the prop.trend.test-function. No adjustment for multiple testing was done. Rates of events in patients with MP are expressed as annual averages in % of patients hospitalized for the main diagnosis of MP. All analyses were performed with GraphPad Prism Version 8, IBM SPSS Statistics version 27, and R Version 4.0.0.

## Results

A mean number of 729 (± 67.5) patients were hospitalized for MP in years 2005, 2007, 2009, 2011, 2013, 2016, 2017, and 2018 in Germany, and a total of 5828 MP patients were included into our final analysis.

### Trends in imaging diagnostics

**Fig. 1:**
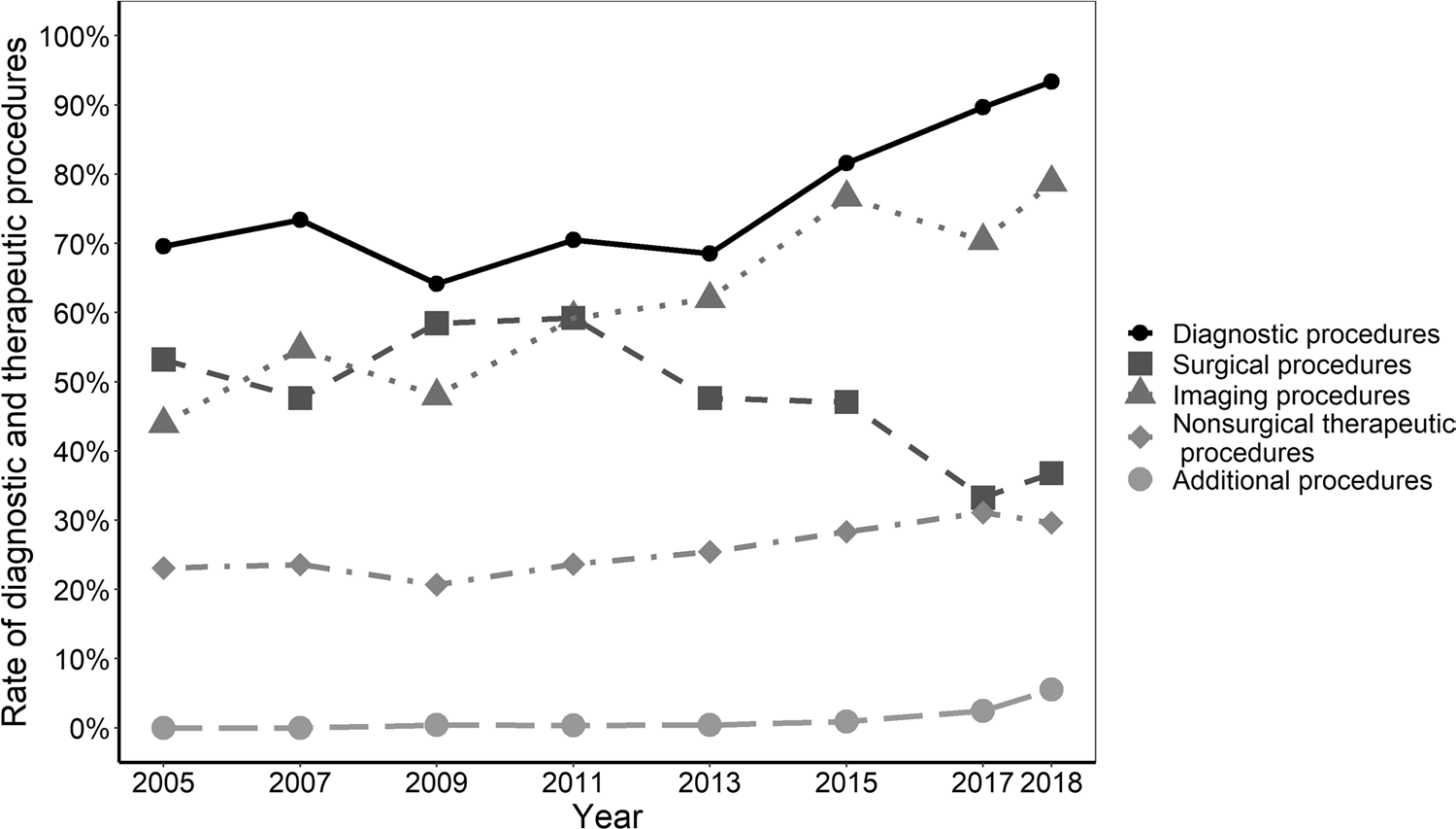
The rate of diagnostic and therapeutic procedures in patients hospitalized for MP. The annual rate of the respective procedure is depicted in % of patients hospitalized for MP. The presence of systematic time trends was investigated with the prop.trend.test-function. Diagnostic, imaging, non-surgical, and additional procedures showed significant increases of rates (*p* < 0.001, respectively) whereas surgical procedure rates decreased (*p* < 0.001). Abbreviations: MP, meralgia paresthetica

**Fig. 2:**
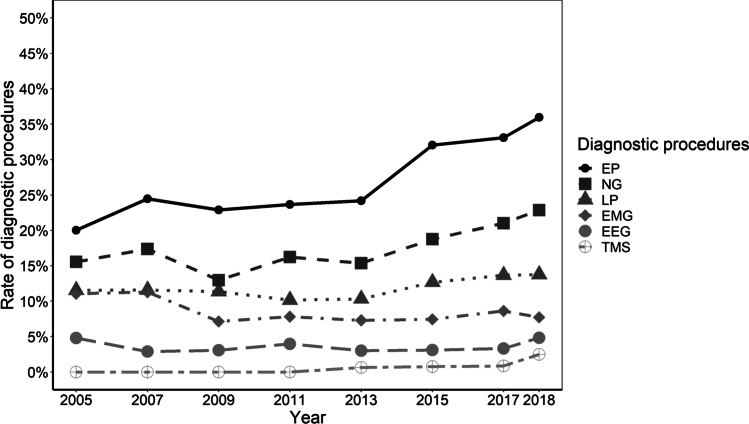
The type of diagnostic procedures in patients hospitalized for MP. The annual rate of the respective procedure is depicted in % of patients hospitalized for MP. The presence of systematic time trends was investigated with the prop.trend.test-function. Rates of NG, EP, and TMS increased (*p* < 0.001, respectively) whereas the rate of EMG decreased (*p* = 0.009). For better readability, this figure displays neurophysiological diagnostics, only. Mean rates of other diagnostic procedures like biopsy, endoscopy, physiologic function tests, and procedures that were not further specified were 1% (± 0.4), 4% (± 1), 1% (± 0.4), and 1% (± 1), respectively. Abbreviations: EP, evoked potentials; NG, neurography; LP, lumbar puncture; EMG, electromyography; EEG, electroencephalography; MP, meralgia paresthetica; TMS, transcranial magnetic stimulation

The rate of imaging studies increased from 44 (*n* = 274) to 79% (*n* = 572) (*p* < 0.001), with a corresponding annual average of 62% (± 13) (Fig. 1). Throughout the entire study period, the mean rate of computed tomography diagnostics was 18% (± 3), compared to 37% (± 8) for magnetic resonance imaging. Trendwise, there was a slight increase in the rates of CT imaging, from 16% (*n* = 101) in 2005 to 22% (*n* = 160) in 2018 (*p* < 0.001), compared to a more pronounced increase in rates of MRI from 26 (*n* = 165) to 45% (*n* = 328) (*p* < 0.001) (Fig. 2). MRI of the spine was predominantly performed in our patient cohort, followed by MRI of the pelvis (Supplemental Table 1 presents a detailed depiction of CT and MRI imaging specificities).

### Trends in non-imaging diagnostics

**Fig. 3:**
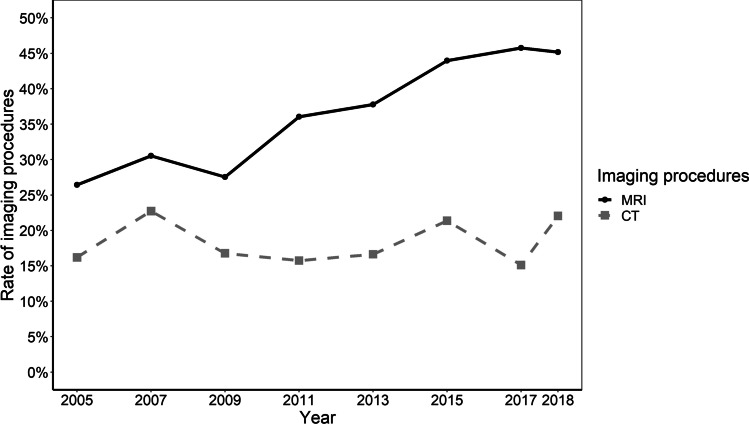
The type of cross-sectional imaging procedures in patients hospitalized for MP. The annual rate of the respective procedure is depicted in % of patients hospitalized for MP. The presence of systematic time trends was investigated with the prop.trend.test-function. Rates of CT and MRI increased significantly over time (*p* < 0.001, respectively). Abbreviations: CT, computer tomography; MP, meralgia paresthetica; MRI, magnetic resonance imaging

Among patients hospitalized for MP, the rate of non-imaging diagnostic studies increased from 70% (*n* = 434) in 2005 to 93% (*n* = 678) in 2018 (*p* < 0.001) with an annual average of 69% (± 11) (Fig. 1). The average annual rate of electrophysiological studies throughout the study period was 57% (± 9) (electroencephalography: 4% (± 1), neurography: 18% (± 3), electromyography: 9% (± 2), and evoked potentials: 27% (± 6)). Between 2005 and 2018, rates of evoked potentials and neurography procedures increased from 20%/16% (*n* = 125/*n* = 97) to 36%/23% (*n* = 261/*n* = 166) (*p* < 0.001, respectively). Rates of electromyography decreased from 11 (*n* = 69) to 8% (*n* = 56) (*p* = 0.009). Transcranial magnetic stimulation (TMS) was first performed in 2013, at a rate of 1% (*n* = 5), increasing to 3% (*n* = 18) in 2018 (*p* < 0.001). The average annual prevalence of lumbar puncture in MP patients was 12% (± 1) without significant changes between 2005 (12%/*n* = 72) and 2018 (14%/*n* = 100) (*p* = 0.056) (Fig. 3).

### Trends in MP treatment

**Fig. 4:**
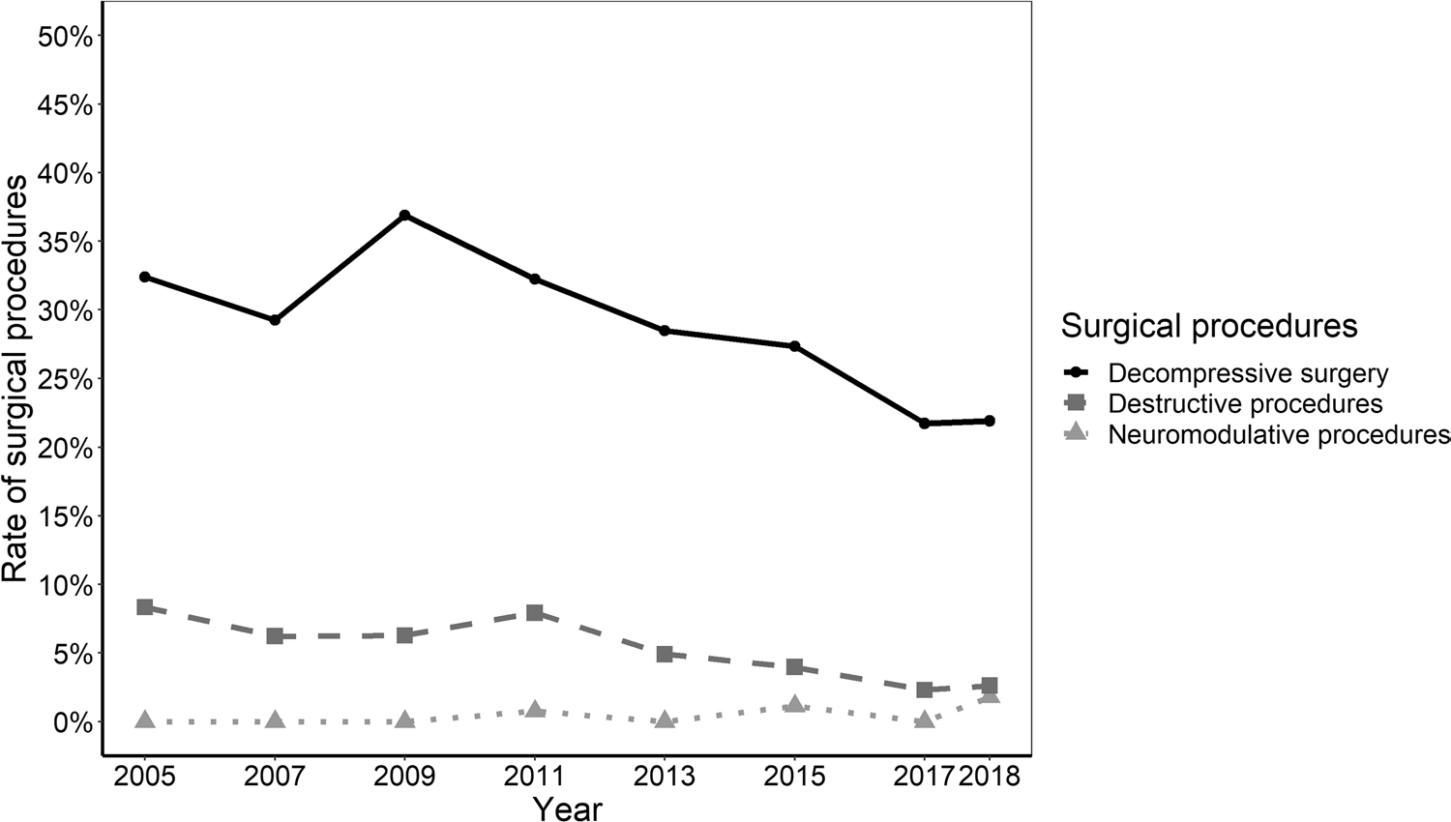
The type of surgical procedures in patients hospitalized for MP. The annual rate of the respective procedure is depicted in % of patients hospitalized for MP. The presence of systematic time trends was investigated with the prop.trend.test-function. Rates of decompressive and nerve transection procedures decreased significantly over time whereas neuromodulative procedures increased (*p* < 0.001, respectively). Abbreviations: MP, meralgia paresthetica

The rates of in-hospital surgical procedures for MP decreased from 53% (*n* = 332) in 2005 to 37% (*n* = 243) in 2018 (mean 48% ± 9, *p* < 0.001), while non-surgical procedures (spinal and local injections, physiotherapy, electrotherapy) increased from 23 (*n* = 144) to 30% (*n* = 215) (mean 26% ± 4, *p* < 0.001). Additional therapies, such as the treatment of psychosomatic and psychic components of MP, increased from 0 (*n* = 0) to 6% (*n* = 40), with a mean annual rate of 1.3% (± 2; *p* < 0.001) (Fig. 1). Throughout the entire study period, the most frequent surgical interventions were decompressive procedures, with an annual average rate of 29% (± 5), but a decrease over time from 32% (*n* = 202) in 2005 to 22% (*n* = 159) in 2018 (*p* < 0.001). Rates of nerve transection procedures at the LFCN ranged substantially lower, at an annual average of 5% (± 2), and also showed a decreasing trend from 8 (*n* = 52) to 3% (*n* = 19) (*p* < 0.001). Neuro-modulative procedures were first performed for MP in 2011 at a rate of 0.7% (*n* = 6), increasing to 1.8% (*n* = 13) in 2018 (*p* < 0.001) (Fig. 4).

## Discussion

This is the first comprehensive analysis of trends in diagnostics and treatment of MP on a national level. The main results are, that diagnostic procedures, both imaging and non-imaging, became more prevalent between 2005 and 2018, while rates of surgical treatment decreased over time, with neuropreservative surgical techniques being performed substantially more frequently than techniques of nerve transection.

The fact that, in Germany, health care professionals increasingly rely on imaging, evoked potentials, and neurography in the diagnosis of MP is especially interesting, since the diagnosis of MP is based first and foremost on clinical examination. Increasing rates of MRI may, in parts, be due to increased incentive to exclude lumbar radiculopathy, given that delayed or false diagnoses comprise about 30% of litigation claims in neurosurgery [30, 31]. Growing rates of MRI in MP diagnostics may also be due to the more widespread availability of MRI and improvements in nerve visualization. This trend could lead to even higher rates of MRI in MP management in the future [32–36]. The fact that rates of other diagnostics, such as LFCN conduction studies [7, 37, 38] and somatosensory evoked potentials, have also increased over time, may reflect increasingly robust evidence on their merits in MP diagnostics [12, 13]. For example, a study from 2006 found that changes in ipsilateral somatosensory evoked potentials (SSEPs) after stimulation of the posterior tibial nerve showed good sensitivity and specificity (85.7% and 82.4%, respectively; accuracy, 83.3%) for MP without the need for bilateral comparisons [14].

Regarding trends in MP management, we found that rates of surgical procedures decreased. At the same time, rates of non-surgical treatments and additional procedures focussing on psychosomatic components increased. Decreasing rates of surgical therapy in MP may be explained by improved medical treatment, such as anti-neuropathic pain medication, which may allow patients and therapists to forgo surgery [39, 40]. Another reason may be that a certain proportion of MP surgery may increasingly take place in an ambulatory setup. For example, in other nerve compression syndromes, such as cubital tunnel syndrome, a trend toward outpatient surgical management has been reported [41, 42]. To date, no data on shifts in the management of MP from in-hospital to outpatient environments exist. Therefore, it is unclear how many additional patients are operated on in the outpatient setting. However, even if we cannot assess potential changes in total numbers, we do not expect the proportions of types of surgery to change.

Throughout the entire study period, surgical management of MP in Germany was conducted using neuropreservative techniques, such as decompression of the LFCN, rather than neurectomy procedures. In the ongoing discussion on which surgical strategy to choose, the most comprehensive meta-analysis had to rely on observational studies in the absence of randomized controlled data [29]. Similar to our findings, it reports that neurolysis was more common than neurectomy. Regarding outcomes, complete pain relief was achieved more often after neurectomy (85%) than decompression surgery (63%). These results are supported by two Cochrane meta-analyses, who report slightly higher rates of postoperative benefit after neurectomy (94%) than after decompression surgery (88%) [19, 20]. Surgeons preferring neurectomy over neurolysis frequently point to histopathological findings in the compressed nerve. These findings suggest focal demyelination, thickened perineurium, subperineurial edema, Renaut bodies, and regenerating clusters [43, 44]. One may argue that such morphological changes are irreversible and neurectomy may therefore represent a more effective surgical strategy in MP than neurolysis [44]. Findings in animal models suggest a relationship between the duration of symptoms and reversibility of ischemic damage to the nerve induced by compression, yet clinical data on the reversibility of histopathological changes in nerve compression syndromes in humans is missing [45, 46]. In a recent observational study, hypesthesia in the innervation area of the LFCN after neurectomy was described as not “bothersome,” as measured on a bothersomeness scale ranging from 0 to 6 [26]. However, a systematic analysis of the effect of anterolateral thigh hypesthesia based on the validated quality-of-life measures is still lacking. A recent description of a more extensive and dynamic decompression technique revealed excellent results in terms of improvement of pain and/or paresthesia (89%), complete restoration of sensory function (69%), and some sensory improvements (26%) [27]. Transposition is a technical variation, which was shown to improve outcomes of neurolysis of the LFCN [28].

Increases in rates of complementary treatment modalities for chronic pain, such as treatment of psychosomatic components, as observed in our analysis in Germany, may reflect changes in chronic pain management toward more holistic approaches. Nevertheless, reports for their particular benefits in MP are lacking [47–49].

A major strength of our study is that it allows for a comprehensive nationwide assessment across all disciplines involved in in-hospital MP care. However, our study has certain limitations. The fact that only data from Germany were included may limit the generalizability of our findings to healthcare systems in other countries. Also, the data set used was depersonalized, and therefore, distinct per-person analyses were not possible. Also, we cannot exclude the possibility of multiple inclusions per person. As mentioned above, patients treated in the outpatient sector were not included in our analysis, potentially introducing a certain degree of selection bias. However, surgical MP treatment in Germany is only rarely conducted in ambulatory facilities, and outpatient data on MP are not collected in a centralized system, unlike in-hospital data, which are more reliable in the assessment of treatment trends [42].

## Conclusions

Between 2005 and 2018, MP care in Germany underwent significant changes. Rates of imaging, evoked potentials, neurography, and non-surgical management increased, while rates of surgical management decreased. Nerve transections for surgical treatment of MP were substantially less frequent than decompressive techniques.

### Supplementary Information

Below is the link to the electronic supplementary material.

Supplementary file1 Supplemental Table Total numbers of specific imaging locations. Total numbers of specific imaging locations are depicted for the respective years. *Abbreviations*: *CT* – *computertomography*, *MRI* –* magnetic resonance image*. (DOCX 20 KB)

## Data Availability

Data is available on reasonable request.
