# Supplementary material for: Diagnosis and treatment of meralgia paresthetica between 2005 and 2018: a national cohort study
Source: Neurosurg Rev. 2023 Feb 13;46(1):54. doi: 10.1007/s10143-023-01962-0 (PMC9925535; doi:10.1007/s10143-023-01962-0)
Supplement: Supplementary file 1 — Supplementary file1 Supplemental Table Total numbers of specific imaging locations. Total numbers of specific imaging locations are depicted for the respective years. Abbreviations: CT – computertomography, MRI – magnetic resonance image. (DOCX 20 KB) [file 10143_2023_1962_MOESM1_ESM.docx]

**Supplemental Table 1**

|  |  | **total numbers** | | | | | | | |
| --- | --- | --- | --- | --- | --- | --- | --- | --- | --- |
| **CT** | Code | **2005** | **2007** | **2009** | **2011** | **2013** | **2015** | **2017** | **2018** |
| CT neurocranium | 3-200 | 20 | 25 | 31 | 24 | 41 | 36 | 38 | 53 |
| CT throat | 3-201 | 0 | 0 | 0 | 0 | 0 | 0 | 0 | 0 |
| CT thorax | 3-202 | 0 | 0 | 0 | 0 | 0 | 0 | 0 | 3 |
| CT spine | 3-203 | 32 | 45 | 30 | 34 | 24 | 33 | 31 | 29 |
| CT heart | 3-204 | 0 | 0 | 0 | 0 | 0 | 0 | 0 | 0 |
| CT skelettal system | 3-205 | 3 | 5 | 3 | 0 | 3 | 5 | 6 | 6 |
| CT pelvis | 3-206 | 7 | 9 | 6 | 9 | 14 | 15 | 10 | 9 |
| CT abdomen | 3-207 | 6 | 9 | 8 | 8 | 6 | 9 | 0 | 4 |
| CT peripheral vessels | 3-208 | 0 | 0 | 0 | 0 | 0 | 0 | 0 | 0 |
| CT neurocranium with contrast | 3-220 | 4 | 0 | 0 | 3 | 0 | 0 | 0 | 4 |
| CT throat with contrast | 3-221 | 0 | 0 | 0 | 0 | 0 | 0 | 0 | 3 |
| CT thorax with contrast | 3-222 | 0 | 3 | 0 | 3 | 4 | 8 | 0 | 4 |
| CT spine with contrast | 3-223 | 0 | 6 | 3 | 0 | 0 | 4 | 0 | 0 |
| CT heart with contrast | 3-224 | 0 | 0 | 0 | 0 | 0 | 0 | 0 | 0 |
| CT abdomen with contrast | 3-225 | 17 | 38 | 23 | 35 | 27 | 38 | 16 | 26 |
| CT pelvis with contrast | 3-226 | 12 | 17 | 11 | 11 | 13 | 21 | 4 | 14 |
| CT skelettal system with contrast | 3-227 | 0 | 0 | 0 | 0 | 0 | 3 | 0 | 0 |
| CT peripheral vessels with contrast | 3-228 | 0 | 0 | 0 | 0 | 0 | 0 | 0 | 5 |
|  |  |  |  |  |  |  |  |  |  |
| **MRI** |  |  |  |  |  |  |  |  |  |
| MRI throat | 3-801 | 0 | 0 | 0 | 3 | 0 | 0 | 0 | 0 |
| MRI spine | 3-802 | 75 | 99 | 76 | 119 | 116 | 139 | 133 | 130 |
| MRI heart | 3-803 | 0 | 0 | 0 | 0 | 0 | 0 | 0 | 0 |
| MRI abdomen | 3-804 | 0 | 0 | 0 | 0 | 0 | 3 | 0 | 0 |
| MRI pelvis | 3-805 | 20 | 26 | 10 | 30 | 27 | 32 | 31 | 31 |
| MRI skelettal system | 3-806 | 4 | 11 | 13 | 18 | 18 | 16 | 14 | 16 |
| MRI breast | 3-807 | 0 | 0 | 0 | 0 | 0 | 0 | 0 | 0 |
| MRI peripheral vessels | 3-808 | 0 | 0 | 0 | 0 | 7 | 0 | 4 | 3 |
| MRI thorax | 3-809 | 0 | 0 | 0 | 0 | 0 | 0 | 0 | 0 |
| MRI neurocranium with contrast | 3-820 | 13 | 19 | 19 | 25 | 27 | 30 | 31 | 30 |
| MRI heart with contrast | 3-821 | 0 | 0 | 0 | 0 | 0 | 0 | 0 | 0 |
| MRI thorax with contrast | 3-822 | 0 | 0 | 0 | 0 | 0 | 0 | 0 | 0 |
| MRI spine with contrast | 3-823 | 19 | 9 | 25 | 32 | 28 | 39 | 37 | 35 |
| MRI heart with contrast | 3-824 | 0 | 0 | 0 | 0 | 0 | 0 | 0 | 0 |
| MRI abdomen with contrast | 3-825 | 5 | 6 | 3 | 0 | 4 | 4 | 0 | 3 |
| MRI skelettal system with contrast | 3-826 | 4 | 10 | 5 | 8 | 7 | 10 | 4 | 5 |
| MRI breast with contrast | 3-827 | 0 | 0 | 0 | 0 | 0 | 0 | 0 | 0 |
| MRI peripheral vessels with contrast |  | 0 | 3 | 0 | 0 | 3 | 0 | 3 | 0 |
| MRI pelvis with contrast | 3-82a | 9 | 10 | 12 | 17 | 18 | 30 | 17 | 14 |
| MRI ventriculography | 3-840 | 0 | 0 | 0 | 0 | 0 | 0 | 0 | 0 |
| MRI myelography | 3-841 | 0 | 0 | 4 | 4 | 5 | 7 | 0 | 10 |
| MRI sialography | 3-842 | 0 | 0 | 0 | 0 | 0 | 0 | 0 | 0 |
| MRI cholangiopancreaticography [MRCP] | 3-843 | 0 | 0 | 0 | 0 | 0 | 0 | 0 | 0 |
| MRI arthrocraphy | 3-844 | 0 | 0 | 0 | 0 | 0 | 0 | 0 | 0 |
| MRI elastography | 3-845 | 0 | 0 | 0 | 0 | 0 | 0 | 0 | 0 |
| MRI liver for iron determination | 3-846 | 0 | 0 | 0 | 0 | 0 | 0 | 0 | 0 |
